# UDP-glucuronosyltransferases and biochemical recurrence in prostate cancer progression

**DOI:** 10.1186/s12885-017-3463-6

**Published:** 2017-07-03

**Authors:** Delores J. Grant, Zinan Chen, Lauren E. Howard, Emily Wiggins, Amanda De Hoedt, Adriana C. Vidal, Skyla T. Carney, Jill Squires, Clara E. Magyar, Jiaoti Huang, Stephen J. Freedland

**Affiliations:** 1 Department of Biological and Biomedical Science, Cancer Research Program, North Carolina Central University, Julius L. Chambers Biomedical/Biotechnology Research Institute, 1801 Fayetteville Street, Durham, NC 27707 USA; 2 Department of Biostatistics and Bioinformatics, Duke University Medical Center, 2424 Erwin Road, Suite 1102 Hock Plaza, Box 2721, Durham, NC 27710 USA; 3 Durham Veterans Administration Medical Center, 508 Fulton St, Durham, NC 27705 USA; 4 Cedars-Sinai Health System, Center for Integrated Research on Cancer and Lifestyle, Cancer Genetics and Prevention Program, Surgery, 8700 Beverly Blvd, Los Angeles, CA 90048 USA; 5 Department of Pathology and Laboratory Medicine, University of California at Los Angeles, The David Geffen School of Medicine at UCLA, 10833 Le Conte Avenue, CHS 14-112, Los Angeles, CA 90095 USA

**Keywords:** Prostate cancer, Biochemical recurrence, UDP-glucuronosyltransferases, UGT2B17

## Abstract

**Background:**

Uridine 5′-diphosphate-glucuronosyltransferase 2B (UGT2B) genes code for enzymes that catalyze the clearance of testosterone, dihydrotestosterone (DHT), and DHT metabolites in the prostate basal and luminal tissue. The expression of the UGT2B15, UGT2B17, and UGT2B28 enzymes has not been evaluated in prostate tissue samples from hormone therapy-naïve patients.

**Methods:**

We determined the expression of UGT2B15, UGT2B17, and UGT2B28 enzymes in 190 prostate tissue samples from surgical specimens of a multiethnic cohort of patients undergoing radical prostatectomy at the Durham Veterans Affairs Medical Center. The association between each protein’s percent positive and H-score, a weighted score of staining intensity, and the risk of biochemical recurrence (BCR) was tested using separate Cox proportional hazards models. In an exploratory analysis, UGT2B17 total positive and H-score were divided at the median and we tested the association between UGT2B17 group and risk of BCR.

**Results:**

The median follow-up for all patients was 118 months (IQR: 85-144). Of 190, 83 (44%) patients developed BCR. We found no association between UGT2B15 or UGT2B28 and risk of BCR. However, there was a trend for an association between UGT2B17 and BCR (HR = 1.01, 95% CI 1.00-1.02, *p* = 0.11), though not statistically significant. Upon further investigation, we found that patients with UGT2B17 higher levels of expression had a significant increased risk of BCR on univariable analysis (HR = 1.57, 95% CI 1.02-2.43, *p* = 0.041), although this association was attenuated in the multivariable model (HR = 1.50, 95% CI 0.94-2.40, *p* = 0.088).

**Conclusions:**

Our findings suggest that UGT2B17 overexpression may be associated with a significant increased risk of BCR. These results are consistent with previous reports which showed UGT2B17 significantly expressed in advanced prostate cancer including prostate tumor metastases.

## Background

It is estimated that there will be approximately 161,360 new cases of prostate cancer (PC) and approximately 26,730 deaths from PC in the US population in 2017 [1]. While overall the average annual percent change in PC incidence and death from 2009 to 2013 has decreased by 8.6%, the death rates in black men was 2.3 times those in white men. Critical to the treatment and prognosis of prostate cancer is the identification of biomarkers by tumor clinicopathologic characteristics to direct the development of therapies and treatment options. Biomarkers that are involved in adrenal and instraprostatic androgen biosynthesis and metabolism have emerged as important targets [2]. Common androgen deprivation therapy (ADT) agents used in individuals with metastatic prostate cancer point to the relevance of androgens/androgen receptor signaling in tumor progression in the prostate cancer cell microenvironment [3]. Various expression levels of steroideogenic enzymes, involved in the biosynthesis of testosterone precursors, have been differentially associated with prostate tumor progression to castration-resistant prostate cancer (CRPC) [2, 4–6]. Moreover, recent evidence suggests that steroideogenic enzymes responsible for the catabolism of intraprostatic testosterone metabolites may be linked to biochemical recurrence (BCR) and tumor progression to CRPC, however, those associations are less understood [2, 7–12].

**Fig. 1:**
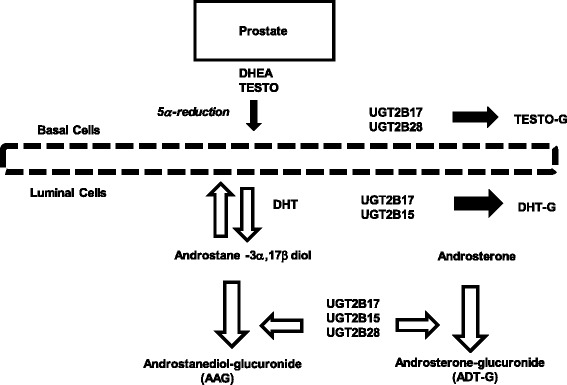
Glucuronidation targets for UGT2B15, UGT2B17, and UGT2B28 enzymes in the prostate [3, 19, 23]. Thick black arrows indicate irreversible production of testosterone- and DHT-G. Curved arrow indicates irreversible production of ADT-G; and parallel wide arrow indicates irreversible production of AAG. DHEA, dehydroepiandersterone; Testo, testosterone; DHT, dihydrotestosterone; ADT-G, androsterone glucuronide

Uridine 5′-diphosphate-glucuronosyltransferases (UGT) are part of a superfamily of enzymes which are part of the phase II drug detoxification pathways in the hepatic tissue in mammals. The UGTs catalyze the addition of the hydrophilic moiety, glucuronide to acceptor molecules in a process called glucuronidation [13, 14]. In humans there are two major classes, UGT1 and UGT2, which each contain multiple genes on chromosome 2 and 4, respectively. The UGT2B subfamily is comprised of *UGT2B4*, *UGT2B7*, *UGT2B11*, *UGT2B15*, *UGT2B17*, and *UGT2B28* along with six pseudogenes that are interspersed among the genes in the region [15]. Enzymes UGT2B15 and UGT2B17 exhibit substrate specificity for androgens such as testosterone, dihydrotestosterone (DHT), DHT metabolites, androsterone and 5α-androstane-3α,17β-diol [16, 17]. Furthermore, those enzymes conjugate androgens present in the lumen and basal epithelial tissue of the prostate [18, 19]. The UGT2B28 enzymes have been shown to be expressed in human testis, prostate, and prostate cancer cell line, LNCaP, where they conjugate 5α-androstane-3α,17β-diol, 5β-androstane-3α,17β-diol and androsterone, and testosterone [20] (Fig. 1). The conjugation of androgens and their metabolites by the UGT2B enzymes in prostate tissue is an irreversible reaction, effectively regulating the levels of DHT available for androgen receptor signaling [19, 21]. Thus the expression levels of UGT2B17, B15, and B28 may be predictors of intraprostatic levels of androgens and prostate cancer phenotype.

A couple of studies have suggested that expression levels of the UGT2B17, B15, and 28 enzymes may be prognostic for clinicopathologic characteristics of prostate cancer. In one study the expression levels of UGT2B17 and B15 in prostate tumor samples from ADTh treated patients were compared to expression levels in patients that were untreated or diagnosed as benign prostatic hyperplasia (BPH) [22]. The results showed that UGT2B15 expression in patients that were treated up to 12 months with ADTh was significantly higher when compared to expression levels in patients that received no treatment for a comparable time or were diagnosed with BPH. Results from the same study showed that UGT2B17 expression was significantly elevated for only 5 months in ADTh treated patients. In another study examining the association of UGT2B28 expression, Belledant et al. [21] demonstrated that higher cytoplasmic expression of the protein was significantly associated with higher Gleason scores and positive nodes. In contrast, lower nuclear expression was associated with decreased PSA and positive margins. These studies suggest that UGT2B expression was altered by intraprostatic levels of androgens and tumor aggression.

To assess whether expression levels of UGT2B enzymes would predict worse prostate cancer outcomes, we analyzed the association between expression of UGT2B15, B17 and B28 in tumor microarrays at the time of radical prostatectomy and risk of BCR. We hypothesized that higher expression of the UGT2B enzymes is associated with increased prostate cancer progression risk.

## Methods

### Study design

A retrospective study was conducted on 196 patients undergoing radical prostatectomy at the Durham Veterans Affairs Medical Center (DVAMC) between 1993 and 2004. None of the patients had hormonal ablation or radiation therapy prior to surgery. Four core needle biopsies of cancer of the formalin fixed and paraffin embedded radical prostatectomy specimens were used to create tissue microarrays (TMAs). Slices of the TMA were stained for expression of UGT2B15, UGT2B17, and UGT2B28. We used two indicators for the staining power of the biomarkers, the average percentage of positive staining cells, and H-score. H-score was calculated for each of the three biomarkers using the formula: 3*percent staining strong +2*percent staining medium +1*percent staining weak staining.

Patients were followed for a median of 118 months. Biochemical recurrence (BCR) was defined as a single PSA value of >0.2 ng/ml, two values of 0.2 ng/ml, or need for secondary treatment for elevated PSA in the post-operative period. We excluded 1 patient missing extra-capsular extension, 2 missing seminal vesicle, and 3 missing follow-up, leaving a total of 190 patients in our study cohort. Institutional Review Board approval was obtained at Duke University, North Carolina Central University and the DVAMC and all patients signed an informed consent at the DVAMC prior to enrollment.

#### Immunohistochemistry of tissue microarrays

For all antibodies, TMA slides from DVAMC were baked overnight in a 60 °C oven before IHC staining. The staining protocols are as follows: UGT2B15: Sections were deparaffinized with xylene and rehydrated through graded ethanol. Endogenous peroxidase activity was blocked with 3% hydrogen peroxide in methanol for 10 min. Heat-induced epitope retrieval (HIER) was carried out in EDTA, pH 8.0 (Invitrogen, cat# 005501) using a vegetable steamer at 95 °C for 25 min. Mouse polyclonal anti-human UGT2B15 primary antibody (Abcam, ab89274) was diluted with BSA to a concentration of 1:50 and applied to the sections overnight at 4 °C. Slides were rinsed with PBS then Dakocytomation Envision System Labelled Polymer HRP anti-mouse (DakoCytomation, cat# K4001) secondary antibody was applied for 30 min at room temperature. The antibody was then visualized using the Betazoid DAB Chromogen Kit (BioCare Medical, cat# BDB2004L). Finally, sections were rinsed with water, counterstained with hematoxylin, dehydrated through graded ethanol, cleared with xylene, and coverslipped.

UGT2B17: Sections were deparaffinized with xylene and rehydrated through graded ethanol. Endogenous peroxidase activity was blocked with 3% hydrogen peroxide in methanol for 10 min. Heat-induced epitope retrieval (HIER) was carried out in EDTA, pH 8.0 (Invitrogen, cat# 005501) using a vegetable steamer at 95 °C for 25 min. Rabbit polyclonal anti-human UGT2B17 primary antibody (Abcam, ab92610) was diluted with BSA to a concentration of 1:50 and applied to the sections overnight at 4 °C. Slides were rinsed with PBS then Dakocytomation Envision System Labelled Polymer HRP anti-rabbit (DakoCytomation, cat# 4003) secondary antibody was applied for 30 min at room temperature. The antibody was then visualized using the Betazoid DAB Chromogen Kit (BioCare Medical, cat# BDB2004L). DAB was rinsed away with distilled water, cupric sulfate was applied for 5 min, and the tissue was rinsed again with distilled water. Finally, sections were rinsed with water, counterstained with hematoxylin, dehydrated through graded ethanol, cleared with xylene, and coverslipped.

UGT2B28: Sections were deparaffinized with xylene and rehydrated through graded ethanol. Endogenous peroxidase activity was blocked with 3% hydrogen peroxide in methanol for 10 min. Heat-induced epitope retrieval (HIER) was carried out in 0.01 M citrate buffer, pH 6.0 using a vegetable steamer at 95 °C for 25 min. Rabbit polycloncal anti-human UGT2B28 primary antibody (Abcam, ab156131) was diluted with BSA to a concentration of 1:50 and applied to the sections for 45 min at room temperature. Slides were rinsed with PBS then Dakocytomation Envision System Labelled Polymer HRP anti-rabbit (DakoCytomation, cat# 4003) secondary antibody was applied for 30 min at room temperature. The antibody was then visualized using the Betazoid DAB Chromogen Kit (BioCare Medical, cat# BDB2004L). Finally, sections were rinsed with water, counterstained with hematoxylin, dehydrated through graded ethanol, cleared with xylene, and coverslipped.

#### Image analysis

Slides were digitized on a ScanScope AT (Leica Biosystems, Inc., Vista, CA) and morphimetric analysis performed with *Definiens’* Tissue Studio (Definiens Inc., Parsippany, NJ) to determine the percentage of UGT2B15, UGTB17, UGTB28 and EGFR positive cells in a non-biased method. Briefly, stain specific algorithims were created using the pre-defined cytoplasmic detection module and classification tool, positive and negative stained cells within each tissue region were identified. Thresholds were set to classify hematoxylin stain for nuclei and DAB stain for positive cytoplasmic staining. The data were exported to Excel for further statistical analysis. Scanning and analyses were performed through the Translational Pathology Core Laboratory, Department of Pathology and Laboratory Medicine, David Geffen School of Medicine at UCLA.

#### Statistical analysis

Patient characteristics were summarized using median, 25th percentile, and 75th percentile for continuous variables and count and percentage for categorical variables. We tested the association between each biomarker’s (UGT2B15, UGT2B17, UGT2B28 – all continuous) percent positive and H-score and the risk of BCR using separate Cox proportional hazards models. Both crude models and models adjusted for PSA (continuous, log-transformed), age (continuous), pathological Gleason score (2-6 vs. 3 + 4 vs. ≥4 + 3), race (black vs. non-black), positive surgical margins (yes vs. no), extracapsular extension (yes vs. no), and seminal vesicle invasion (yes vs. no) were fit. In these models, UGT2B15, UGT2B17, and UGT2B28 percent positive and H-score were modeled per 10-unit increase. In an exploratory analysis, we split UGT2B17 total positive and H-score at the median and tested the association between UGT2B17 group and risk of BCR.

We also tested whether UGT2B15, UGT2B17, or UGT2B28 H-scores were correlated with pathological Gleason score using the Kruskal-Wallis test or chi-square test. For each level of Gleason score (2-6 vs. 3 + 4 vs. ≥4 + 3), we reported median, 25th percentile, 75th percentile of the biomarker H-score, <median (low expression), and ≥median scores (high expression).

## Results

**Fig. 2:**
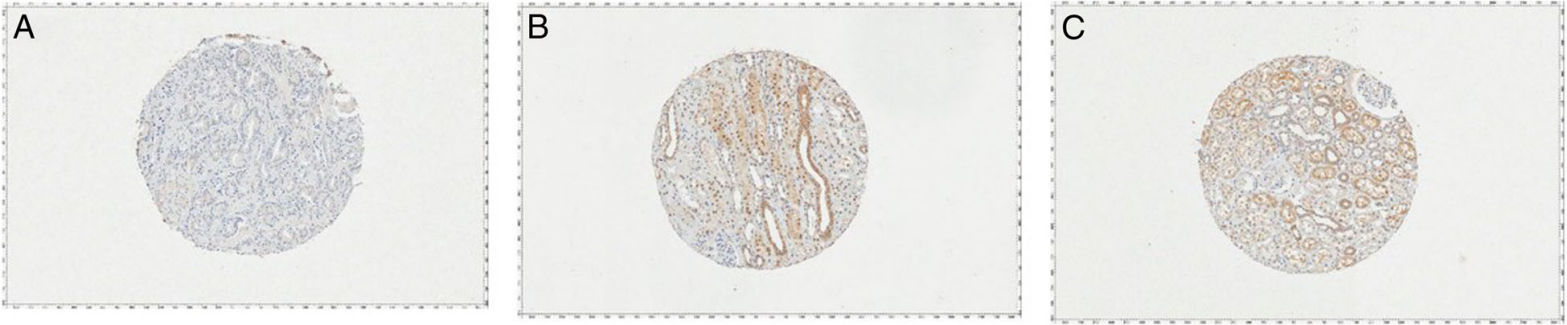
Immunohistochemical detection and specificity of UDP-glucuronosyltransferase 2B (UGT2B) enzymes (brown stain) at 10X magnification. **a** Normal kidney tissue showing negative UGT2B staining. **b** Tonsil tissue showing positive UGT2B staining throughout basal layer. **c** Prostate cancer with positive UGT2B staining

The demographic characteristics of the study population and their clinical and pathologic phenotypes are summarized in Table 1. The median age of the cohort was 63 years (Q1-Q3: 58-67) and less than half (48%) of the patients were black. The median follow-up time was 118 months (Q1-Q3: 85-144 months). Expression of the UGT2B isoforms was assessed by immunohistochemical detection (Fig. 2). The median percentage of cells staining positive for UGT2B15, UGT2B17, and UGT2B28 was 39%, 74%, and 77% respectively. The median of H-score for UGT2B15 was 40, 76 for UGT2B17, and 86 for UGT2B28.

**Table 1:** Demographic, clinical, and pathological characteristics of patients

	All patients (*N* = 190)
Age, M (Q1-Q3)	63 (58-67)
Race, n(%)	
Non-black	99 (52)
Black	91 (48)
Year of Surgery, M (Q1-Q3)	2001 (1997-2002)
PSA (ng/mL), M (Q1-Q3)	7.4 (5.1-11.1)
Pathological Gleason, n(%):	
2 – 6	39 (20)
(3 + 4)	100 (53)
(4 + 3) – 10	51 (27)
Positive Margins, n(%)	115 (61)
Seminal Vesicle Invasion, n(%)	28 (15)
Extracapsular Extension, n(%)	51 (27)
Positive Lymph Nodes, n(%)	1 (<1)
Average % Cells Positive for UGT2B15, M(Q1-Q3)	39 (24-61)
Average % Cells Positive for UGT2B17, M(Q1-Q3)	74 (59-86)
Average % Cells Positive for UGT2B28, M(Q1-Q3)	77 (60-91)
UGT2B15 H-score, M(Q1-Q3)	40 (24-63)
UGT2B17 H-score, M(Q1-Q3)	76 (59-92)
UGT2B28 H-score, M(Q1-Q3)	86 (64-107)
PSA Follow-up (months), M(Q1-Q3)	118 (85-144)

*SD* standard deviation, *M* median; Q1 25th percentile; Q3 75th percentile; *BMI* body mass index, *PSA* prostate specific antigen

There was no association between UGT2B15 or UGT2B28 percent positive or H-score on univariable or multivariable analysis (all HR = 0.98-1.08, *p* > 0.5; Table 2) when biomarkers were modeled per 10-unit increase. Similarly, there was no association between UGT2B17 H-score on univariable or multivariable analysis (HR 1.05, *p* > 0.2; 1.03, *p* > 0.4). However, there was a trend between higher UGT2B17 percent positive and increased risk of BCR (HR 1.11, 95% CI 0.98-1.25, *p* = 0.09) on univariable analysis, although this did not reach statistical significance. This trend was weakened on multivariable analysis (HR 1.10, 95% CI 0.98-1.25, *p* = 0.11).

**Table 2:** Hazard ratios for risk of biochemical recurrence by UGT2B15, UGT2B17, UGT2B28 measures

	UGT2B15		UGT2B17		UGT2B28	
Predictor	HR (95% CI)	*p*-value	HR (95% CI)	*p*-value	HR (95% CI)	*p*-value
% Positive						
Crude	1.98 (0.90-1.07)	0.70	1.11 (0.98-1.25)	0.09	1.02 (0.92-1.13)	0.70
Adjusted^a^	1.02 (0.93-1.12)	0.72	1.10 (0.98-1.25)	0.11	1.08 (0.96-1.21)	0.21
H-score						
Crude	0.99 (0.91-1.07)	0.80	1.05 (0.97-1.13)	0.21	1.00 (0.94-1.06)	0.89
Adjusted^a^	1.03 (0.94-1.12)	0.57	1.03 (0.96-1.11)	0.42	1.04 (0.97-1.11)	0.26

^a^Adjusted for PSA, age, pathological Gleason score, race, year of surgery, positive margins, extracapsular extension, and seminal vesicle invasion

All biomarkers are modeled per 10-unit increase

Notably in secondary analysis, patients with UGT2B17 total positive above the median had a significant increased risk of BCR on univariable analysis (HR = 1.57, 95% CI 1.02-2.43, *p* = 0.041; Table 3). However, this association was attenuated in the adjusted model (HR = 1.50, 95% CI 0.94-2.40, *p* = 0.088). There was no association between UGT2B17 H-score groups and BCR on univariable (*p* = 0.20) or multivariable (*p* = 0.32) analyses. Upon further inspection, pathological Gleason score and seminal vesicle invasion were the greatest mediators between UGT2B17 and BCR. Specifically, after adjusting for these two variables, the HR is 1.28 (95% CI 0.81-2.00; *p* = 0.29).

**Table 3:** Hazard ratios for risk of biochemical recurrence by UGT2B17 total percent positive and H-score 2 groups

	Crude		Adjusted^a^	
UGT2B17 total positive	HR (95% CI)	*p*-value	HR (95% CI)	*p*-value
Group 1	Reference	-	Reference	-
Group 2	***1.574 (1.018, 2.434)***	***0.041***	1.502 (0.941, 2.398)	0.088
UGT2B17 H-score				
Group 1	Reference	-	Reference	-
Group 2	1.324 (0.859, 2.039)	0.203	1.259 (0.780, 1.983)	0.319

^a^Adjusted for PSA, age, pathological Gleason score, race, year of surgery, positive margins, extracapsular extension, and seminal vesicle invasion

Bold italics denotes statistical significance﻿

Finally, we found no association between H-scores of UGT2B15, UGT2B17, or UGT2B28 and pathological Gleason score (*p* = 0.34, 0.92, 0.80, respectively; Table 4). There were no interactions between the biomarkers and race (*p* = 0.65 for UGT2B15, *p* = 0.10 for UGT2B17, *p* = 0.27 for UGT2B28).

**Table 4:** Association between UGT2B15, UGT2B17, UGT2B28 H-scores and pathological Gleason score

	Pathological Gleason Score			
	2 – 6	3 + 4	(4 + 3) – 10	*P*-value
UGT2B15				0.55*
Median	42.8	39.0	31.3	
Q1-Q3	28.8-61.9	21.5-65.9	24.5-50.5	
< Median	16 (41%)	48 (48%)	31 (61%)	0.15**
≥ Median	23 (59%)	52 (52%)	20 (39%)	
UGT2B17				0.84*
Median	72.4	78.5	78.2	
Q1-Q3	59.8-88.1	58.6-94.9	68.2-84.6	
< Median	22 (56%)	48 (48%)	25 (49%)	0.66**
≥ Median	17 (44%)	52 (52%)	26 (51%)	
UGT2B28				0.89*
Median	86.0	86.0	86.4	
Q1-Q3	58.4-102.9	63.6-109.3	74.1-102.2	
< Median	20 (51%)	50 (50%)	25 (49%)	0.98**
≥ Median	19 (49%)	50 (50%)	26 (51%)	

*P*-value calculated using *Kruskal-Wallis test or **chi-squared test

## Discussion

There is increasing evidence that standard approaches to ADT that involve targeting enzymes involved in androgen biosynthesis and orchiectomy are not sufficient to prevent the development of CRPC [2]. The goal of these therapies is to diminish the availability of DHT activated androgen receptor (AR) to promote cancer cell proliferation and growth through AR specific gene activation [6]. The UGT2B enzymes, UGT2B15, B17, and B28, which are regulated in part by the AR, are responsible for the removal of DHT metabolites in an irreversible glucuronidation reaction [19, 23]. In vitro experimental evidence has shown that the amounts of DHT are lower in culture media in control prostate cancer cell lines not treated with UGT2B17/B15 si-treated cell. Thus the enzymes may play a critical role in hormone levels in prostate cancer influencing tumor aggression and promotion. We sought to evaluate the expression of three UGT2B enzymes for their association with clinical and pathological characteristics of a multiethnic population from the Durham Veterans Affairs Medical Center, and evaluate whether their expression levels would predict prostate cancer outcomes after radical prostatectomy. Our results show that UGT2B17 overexpression was associated with a significant increased risk of BCR and that association was attenuated in adjusted models. That association was not modified by race. However, there were no significant associations between UGT2B17 expression and pathological Gleason score. The expression of UGT2B15 and B17 enzymes was not associated with any clinical and/or pathological characteristics.

To date no study has compared the expression of UGT2B17 and UGT2B17 to clinicopathologic characteristics of prostate tissue. Results from Grosse et al., [22] showed that expression of the UGT2B enzymes in samples from ADT treated patients were significantly higher than those of untreated patients. However UGT2B17 and B15 expression in the ADT untreated samples was not evaluated for association with clinical and pathological characteristics of prostate cancer such as BCR, PSA, pathological Gleason score, positive surgical margins, extracapsular extension and seminal vesicle invasion. The tissue used in this study was from patients who had no hormonal ablation or radiation therapy prior to prostatectomy surgery, therefore any comparison to the previous study may not be accurate. The results from our study showed no significant associations with BCR and Gleason score for UGT2B28 expression. In contrast, previous report showed that UGT2B28 overexpression was associated with lower PSA, high Gleason score, and BCR [21]. In that study, antibody staining was targeted in the basal cells of the prostate tumor and were cytoplasmic or nuclear. Further patient tissue samples with strong nuclear staining were associated with low PSA and positive margins while strong cytoplasmic staining was associated with positive nodal status and higher Gleason score. Interestingly in that same study, prostate secretory cells showed low to moderate staining. The results in our study emanated from cytoplasmic stained patient tissue samples that were not enriched for specifically for prostate basal cells. The population make-up in the studies may be different since the above study included patients from hospitals in Quebec, Canada and our study included Black and non-black patients in equal proportions. It is not clear if individuals of African descent have been characterized for UGT2B28 expression or polymorphisms. These differences in staining approaches and sample population may account for the differences in results.

Genetic studies have demonstrated a relationship between UGT2B genes and clinical and pathologic characteristics of prostate cancer. For example, a study has shown that deletions of *UGT2B17* and *B28* genes were significantly associated with decreased survival in Caucasians and Asians while deletion of only *UGT2B17* was associated with Gleason score in Asians [24]. While significantly lower steroid glucuronides were found in that study, no other association was shown with PSA, Gleason score and tumor node metastasis. The expression levels of the *UGT2B* genes were not observed. Previous studies have shown that low expression levels of UGT2B17/15 in the LNCaP prostate cancer cell line resulted in lower intracellular glucuronide levels [19, 23]. In the current study, the results suggest that UGT2B17 overexpression was associated with BCR, which may be an indication overexpression may be a marker for tumor progression to CRPC independent of intracellular glucuronide levels. Another *UGT* gene has recently been implicated as a probable prognostic marker. Single nucleotide polymorphisms of the *UGT1A* locus (*UGT1A9*, *UGT1A10*) have been significantly associated with decreased survival following BCR [25]. In that same study, some SNPs from the same genes were protective and conferred decreased risk of BCR. Interestingly, the *UGT1A* risk alleles did not significantly change androgen glucuronide levels. UGT1A class expression was shown to be decreased in prostate cancer stem cells and in metastatic CRPC cells while in vitro experiments with LNCaP prostate cancer cell lines suggests this decreased expression is associated with cell survival [26]. This may be an indication that the expression of *UGT2B* genes in the prostate are critical to the hormonal microenvironment.

Our study has some limitations. The small sample size may have limited the power to detect significant associations between UGT2B17 expression and BCR on multivariable analysis. In addition we had a few number of events for CRPC and metastases, thus we could not evaluate the associations between UGT2B expression levels and those long-term prostate cancer outcomes. UGT2B17 expression has been found to be significantly expressed in prostate cancer metastases and CRPC tumors, while UGT2B15 is negatively regulated in those tissues [26]. Elevated circulating testosterone has been associated with UGT2B28 nuclear staining which may decrease expression of UGT2B15 and UGT2B17 since studies show that their expression is elevated in patients and prostate cancer cell cultures treated with antiandrogens [21, 22]. This suggests that other factors may be acting in tissues with deleted UGT2B17 and have higher biochemical recurrence.

## Conclusion

In conclusion, the results from this study are suggestive that UGT2B17 overexpression may be associated with an increased risk of BCR. This study contributes to an emerging field of investigation that seeks to uncover the novel predictors of intraprostatic levels of androgens that persist in CRPC and the subsequent development of prostate tumor metastases [27]. Future analyses with increased sample sizes and data on *UGT2B* gene variants are needed, as those variants continue to show impact on the risk for prostate cancer [28]. The UGT2B enzymes, UGT2B15, B17, and B28 are responsible for much of the catabolism of testosterone and DHT metabolites in the prostate and must be further investigated to assess their importance as putative biomarkers and therapeutic targets.

## Abbreviations

ADT: androgen deprivation therapy
BCR: biochemical recurrence
DHT: dihydrotestosterone
HR: hazards ratio
UGT: UDP-glucuronosyltransferase
